# Diagnostic Performance of Screening Tools for Depressive Symptoms in Vulnerable Older Patients with Cancer Undergoing Comprehensive Geriatric Assessment (CGA): Results from the SCREEN Pilot Study

**DOI:** 10.3390/curroncol30020140

**Published:** 2023-02-02

**Authors:** Laura Tack, Ann-Sophie Maenhoudt, Lore Ketelaars, Jolien De Zutter, Stefanie Pinson, Laura Keunebrock, Lorenz Haaker, Kathleen Deckmyn, Mathilde Gheysen, Cindy Kenis, Hans Wildiers, Laurence Depoorter, Pieter-Jan Geerts, Rebecca Chandler, Tom Boterberg, Patricia Schofield, Christopher N. Parris, Philip R. Debruyne

**Affiliations:** 1 Department of Medical Oncology, OECI-designated Kortrijk Cancer Centre, General Hospital Groeninge, 8500 Kortrijk, Belgium; 2 Department of Human Structure and Repair, Ghent University, 9000 Ghent, Belgium; 3 Department of Psychology, General Hospital Groeninge, 8500 Kortrijk, Belgium; 4 Department of General Medical Oncology, University Hospitals Leuven, 3000 Leuven, Belgium; 5 Department of Public Health and Primary Care, Academic Centre for Nursing and Midwifery, KU Leuven-University of Leuven, 3000 Leuven, Belgium; 6 Department of Geriatrics, General Hospital Groeninge, 8500 Kortrijk, Belgium; 7 Department of Psychiatry, General Hospital Groeninge, 8500 Kortrijk, Belgium; 8 School of Allied Health, Faculty of Health, Education, Medicine and Social Care, Chelmsford CM1 1SQ, UK; 9 School of Nursing and Midwifery, Faculty of Health, University of Plymouth, Plymouth PL4 8AA, UK; 10 Medical Technology Research Centre (MTRC), School of Life Sciences, Faculty of Science and Engineering, Anglia Ruskin University, Cambridge CB1 1PT, UK

**Keywords:** depression, oncogeriatric, older adults with cancer, Patient Health Questionnaire, Geriatric Depression Scale

## Abstract

Depression is a common and disabling disorder in later life, particularly among people with poor physical health. There are many screening tools available that can be used to examine depressive symptoms; however, not all of them may be appropriate or accurate for older adults with cancer. This pilot study was designed to test the diagnostic performance of two screening tools and their short versions in a cohort of vulnerable (G8 score ≤ 14/17) older patients with cancer undergoing comprehensive geriatric assessment (CGA). The prospective analysis covered 50 vulnerable patients with cancer aged ≥70 years. The diagnostic performance of the Geriatric Depression Scale (GDS)-15, GDS-4, Patient Health Questionnaire (PHQ)-9 and PHQ-2 was compared to the ‘gold standard’ Structured Clinical Interview for DSM-5 Disorders (SCID-5-S) depression module A. The sensitivity and specificity in detecting depressive symptoms were the highest in the case of PHQ-2, with an area under the receiver operating characteristic curve (AUROC) of 92.7%. The AUROC for the 9-item version, PHQ-9, was 90.2%. For the GDS-15 and GDS-4, the AUROC was only 56.2% and 62.0%, respectively. The SCREEN pilot study illustrates the potential benefit of using a shorter screening tool, PHQ-2, to identify older patients with cancer who would benefit from a more in-depth emotional evaluation as part of a CGA.

## 1. Introduction

Depression is one of the most frequent causes of emotional distress in older adults and has a prevalence rate of 6% to 25% in older adults with cancer [1,2,3,4]. Depression often results in suffering and distress and is negatively associated with treatment compliance and responses to rehabilitation programmes. This may lead to negative outcomes in a variety of medical conditions, a prolonged duration of hospital stay and increased mortality [1,5]. Overall, depression can act as a significant determinant of quality of life and survival [6].

Despite its significant prevalence, depression continues to be undetected and undertreated in patients with cancer [7]. This may arise from the challenges in distinguishing depressive symptoms from those attributable to cancer and treatment side effects. These overlapping symptoms may include pain, fatigue, insomnia, changes in appetite and anxiety [8]. An additional challenge in the management of depression, is the underutilisation of existing tools to assess and identify depression among cancer patients [9]. These challenges culminate in the adequate detection and treatment of psychiatric problems among cancer patients, particularly among older adults.

The detection of depression can be improved by routine use of validated screening instruments. The Geriatric Depression Scale (GDS) is a screening tool that is widely used to identify depressive symptoms in older adults, which has been translated into several languages and validated in many countries. The original 30-item version (GDS-30) was transformed into various shorter versions, such as the GDS-15 and GDS-4. These are comparatively quicker to perform and easier to administer and can be more readily used for screening [10,11,12]. Previously, guidelines from the National Comprehensive Cancer Network (NCCN) advocated the use of GDS-15 within a Comprehensive Geriatric Assessment (CGA). Yet, since the GDS is only a screening tool, the GDS-15 is often considered to be too time-consuming in clinical practice. Moreover, it contains a number of inappropriate questions that may also lead to the inaccurate assessment of depression in older adults with cancer. The patients may struggle to interpret questions such as ‘Are you afraid that something bad is going to happen to you?’, especially soon after diagnosis. The responses may therefore reflect their current state of mind rather than any underlying depressive symptoms. Therefore, the GDS-15 result may overestimate depression, leading to a false positive screening, as it may not be representative of the true emotional status of an older adult with cancer.

Based on the literature and clinical practice in our hospital, the short-item version of the GDS-15 was included in this pilot study. From the existing versions of the GDS-4, the most established one, developed by D’Ath et al., was selected [12]. The PHQ-9 was also included, utilising the abbreviated form with two items [13,14]. This is a recently developed measure that has been extensively researched, offering a reliable and valid measure of depression severity [15]. Thus, we decided to utilise these four screening tools in order to provide a workflow of an initial assessment with a short-item version. Where necessary, this short-item version of assessment could be followed by an assessment based on the longer version.

Despite the practical benefits of shorter screening tools, they show heterogeneity in their estimates of sensitivity and specificity, often with a low or very low certainty of the evidence. Moreover, they are often not validated in (vulnerable) older populations with cancer (undergoing CGA). This pilot study was performed to examine the screening performance of the GDS-15, GDS-4, PHQ-9 and PHQ-2 compared to the depression module A of the Structured Clinical Interview for DSM-5 Disorders (SCID-5-S), considered the gold standard for the assessment of depression in the general population.

## 2. Materials and Methods

### 2.1. Study Population and Design

The SCREEN pilot study was a single-centre, prospective, open-cohort study. Patients with cancer aged 70 and older who presented at the Organisation-of-European-Cancer-Institutes (OECI)-designated clinical cancer centre of the General Hospital Groeninge, Kortrijk, Belgium, were included between April 2021 and July 2022. The onco-psychologists of the Kortrijk Cancer Centre, hereafter referred to as trained healthcare workers (THCWs), are qualified to conduct the G8 and CGA, which is routine practice at the General Hospital Groeninge. The THCWs were responsible for identifying vulnerable patients receiving cancer treatment who were potentially eligible to participate in the pilot study. The eligible patients had been diagnosed with a histologically confirmed solid tumour or haematological malignancy (any stage and any type of treatment) and had a vulnerable profile, indicated by an abnormal score on the Geriatric-8 screening tool (G8 ≤ 14/17) or based on the clinical judgement of the THCW. All the vulnerable patients were also scheduled for a CGA, generally based on the G8 score but sometimes based on the clinical insight of the THCW claiming that a CGA is required despite a normal G8 score (G8 > 14/17). Patients who had been treated for depressive symptoms in the past and patients who were receiving active treatment for depressive symptoms at the time of the study were excluded, as their responses could bias the study outcomes. Additionally, patients with moderate or severe cognitive deficits based on the Freund Clock Drawing Test (CDT; cut-off ≤ 4) and/or Folstein’s Mini-Mental State Examination (MMSE; cut-off ≤ 23) or those previously diagnosed with dementia were excluded. Finally, patients with an expected life expectancy < 3 months and patients in an end-of-life care setting were also excluded, as depressive symptoms and the prevalence of depression may differ quite substantially between patients in curative versus end-of-life care settings [16,17,18].This study was approved by the local ethics committee of the General Hospital Groeninge (AZGS2021008). The data for the analysis were registered without written consent, since G8 and CGA are routine practice at our hospital. The ethics committee approved the registration of the demographic, oncological and geriatric parameters within the framework of the PROACTIVE (AZGS2012061) and REGERCAN (AZGS2015081) trials. However, written informed consent for inclusion and the registration of data based on PHQ-9 and SCID-5-S depression module A was mandatory.

### 2.2. Data Collection

Five THCWs (A.-S.M., L.K., J.D.Z, S.P. and L.K.) were responsible for identifying vulnerable patients who were potentially eligible to participate in the pilot study. In cases of a positive screening (G8 ≤ 14) or based on the THCW’s clinical insight, a complete CGA (Appendix A, Table A1), including an examination of the emotional status (GDS-15; Appendix A, Table A2), was performed [11]. Once identified as eligible for participation, patients were approached by the research associate (L.T.) who was responsible for explaining the pilot study and obtaining informed consent. In order to compare the screening tools as accurately as possible, the patients’ symptoms of depression were assessed using the PHQ-9 and the SCID-5-S depression module A within two weeks after the GDS-15 was completed within CGA. All the questionnaires were collected on paper by the THCWs or physicians in specialist training (L.H., K.D. and M.G.) and were abstracted by an independent research associate (L.T.).

### 2.3. Instruments

Geriatric Depression Scale: Within the CGA, the affective status of older patients with cancer is routinely assessed by the GDS-15. The GDS-15 questionnaire addresses the depressive symptoms that patients have experienced within the past week (Appendix A, Table A2). The items are rated as 0 (no; symptom absent) or 1 (yes; symptom present) [11]. Although the GDS-15 is not a diagnostic instrument, the cut-off score of 5 indicates the presence of depressive symptoms. This demonstrated good internal consistency with a Cronbach’s α = 0.94 and good concurrent validity with the (0.84) Zung Self-Rating Depression Scale and (0.83) the Hamilton Depression Rating scale [19]. Several short-item versions of the GDS are also in use. However, the reliability and validity of these versions are not supported by evidence at present [20]. In this pilot study, we included the 4-item version developed by D’Ath et al. and determined the diagnostic accuracy by analysing both reported cut-off scores of 1 and 2 [12].

Patient Health Questionnaire: The 9-item version of this depression module (PHQ-9) scores each of the nine DSM-IV criteria and is endorsed by the National Institute for Health and Clinical Excellence (NICE) for use in primary care to measure baseline depression severity and responsiveness to treatment (Appendix A, Table A3). The items are rated as 0 (not at all) to 3 (nearly every day) [13]. Cumulative scores ≤ 4 suggest minimal depression, which may not require treatment. The PHQ-9 has been validated for use in primary care. In addition to enabling criteria-based diagnoses of depressive disorders, the PHQ-9 is also a reliable and valid measure of depression severity [15]. Moreover, there is also a short version of the PHQ-9 consisting of the first two items (PHQ-2) that has been shown to be an effective screener, with 97% sensitivity and 67% specificity [14,21]. The PHQ-2 explores the degree to which an individual has experienced depressed moods and anhedonia over the two weeks prior to the assessment. Its purpose is not to establish a final diagnosis or to monitor depression severity but rather to screen for depression. The recommended cut-off point is a score of 3 or greater [14].

Structured Clinical Interview for DSM-5 Disorders, depression module A: In 2014, DSM-V was developed and divided into the SCID-P for personality disorders and SCID-S for syndromes or major mental disorders. The structured clinical interview is a diagnostic tool used to determine the disorders, and it is designed to be administered by a mental health professional. In view of the complexity and heterogeneity of depressive symptoms in older patients with cancer, we used the SCID-5-S depression module A (depressive episode: present and past) to establish the presence or absence of a depressive disorder [22]. These items address symptoms that must be present for at least two weeks in the month prior to assessment. This depression module has been used as the ‘gold standard’ for depression and the criterion used to test the sensitivity and specificity of the screening tools.

### 2.4. Statistical Analysis

Descriptive statistics were performed to present the clinical and socio-demographic characteristics.

A receiver operating characteristic (ROC) curve was plotted to evaluate the predictive accuracy of the screening tools in determining the presence of depressive symptoms using the gold standard SCID-5-S depression module A. We examined the area under the receiver operating characteristic curve (AUROC) for each measure to determine whether these measures predicted depressive symptoms greater than chance, including confidence intervals (CI) (95%, two-tailed) to describe the uncertainty associated with this estimate.

For each screening tool, we reported the sensitivity, specificity and positive and negative predictive values based on the published cut-off scores for each measure, including the GDS-15 cut-off score of 5, GDS-4 cut-off scores of 1 and 2, the PHQ-9 cut-off score of 5 and the PHQ-2 cut-off scores of 2 and 3. These are the cut-off scores cited in the literature as identifying the presence of depressive symptoms in the general older adult population. We examined whether or not these cut-off scores were comparable to the optimal cut-off scores in the population of older adults with cancer. Appropriate cut-off scores were selected based on the optimal combination of sensitivity and specificity. We also determined the ‘optimal’ cut-off score by calculating the Youden index (J) (i.e., J = maximum [(Sensitivity +Specificity)-1]), which is recognized as one of the more reliable methods of determining an optimal cut-off score compared to the visual inspection of ROC curves. Positive (LR+) and negative (LR−) likelihood ratios were also calculated. The LR+ may serve as an additional indicator of each measure’s ability to accurately predict the presence of depression, with higher values suggesting better concurrent validity.

Finally, *p*-values below 0.05 were considered statistically significant. All statistical analyses were conducted using Microsoft Office Excel 2019 (Microsoft, Inc., Redmond, WA, USA) and IBM SPSS v.28 (SPSS, Inc., Chicago, IL, USA) software.

## 3. Results

### 3.1. Patient Population

Between April 2021 and July 2022, five THCWs performing geriatric assessments as part of routine practice screened older adults with cancer for eligibility. In total, 92 patients were identified as vulnerable and could potentially participate in the trial. However, 3 were excluded because they were already receiving active treatment for depressive symptoms; 2 indicated that they wished to stop all treatment and were thus not eligible for participation; 1 had severe cognitive deficits; 6 refused to participate; 26 had no further appointments planned for active treatment at the day clinic (follow-up only); and 4 passed away before the next appointment. In total, 50 vulnerable older adults with cancer agreed to participate in the trial. Details of the patient characteristics can be found in Table 1.

### 3.2. Predictive Accuracy of the Screening Tools

According to the SCID-5-S depression module A, 18% of the sample met the criteria for depression (n = 9). The 15- and 4-item versions of the GDS, respectively, identified 16% (n = 8) and 8% (n = 4) of the patients as having depressive symptoms. According to the PHQ-9 and PHQ-2, respectively, 48% (n = 24) and 18% (n = 9) were patients with depressive symptoms.

**Figure 1:**
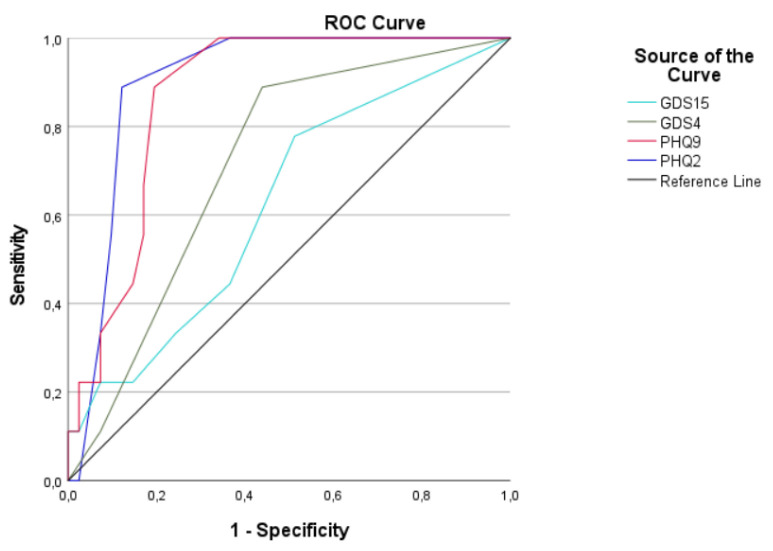
ROC curves of the four depression screening tools according to the gold standard SCID-5-S depression module A. Abbreviations: ROC curve: receiver operating characteristic curve; GDS-15: 15-item version of the Geriatric Depression Scale; GDS-4: 4-item version of the Geriatric Depression Scale; PHQ-9: 9-item version of the Patient Health Questionnaire; PHQ-2: 2-item version of the Patient Health Questionnaire.

The diagnostic performance of the screening tools, according to the gold standard SCID-5-S depression module A, is illustrated in Figure 1.

The AUROC was 62.5% for GDS-15, 71.7% for GDS-4, 87.0% for PHQ-9 and 90.2% for PHQ-2. The details can be found in Table 2.

### 3.3. Diagnostic Performance of the Screening Tools

There were notable differences between the screening tools related to their diagnostic performance at the respective cut-offs. The sensitivity, specificity, false positives (FP), false negative (FN), positive and negative predictive values (PPV and NPV), positive and negative likelihood ratios (LR+ and LR−) and Youden index (J), according to the gold standard SCID-5-S depression module A, are presented in Table 3.

For the GDS-15, the sensitivity was 22.2% and the specificity was 92.7% in detecting depression based on the recommended cut-off score of 5 (Table 3). The ROC analyses and Youden Index revealed that the GDS-15 is not an effective screening tool, according to its diagnostic accuracy.

For the GDS-4, optimal results were obtained using a cut-off score of 1. With a high sensitivity of 88.9% and an NPV of 95.8%, GDS-4 shows characteristics of a good screening instrument. However, the ROC analyses and Youden index indicated that neither of the recommended cut-off scores of 1 and 2 could be used to screen adequately for depression in the population of older patients with cancer (Table 3).

The PHQ-9 obtained an optimal AUROC (Figure 1) and demonstrated an excellent sensitivity and NPV in screening for depressive symptoms based on the recommended cut-off score of 5 (Table 3). Despite these good screening characteristics, the LR+ and Youden index were relatively low.

Although the PHQ-9 has effective screening characteristics, it is the short version of PHQ-2, which obtained better results, as indicated by the ROC analyses (Figure 1; Table 2) and Youden index (Table 3). More optimal results were obtained for the PHQ-2, as a screening instrument, by applying the cut-off score of 2, including a good sensitivity, excellent NPV and the highest Youden index and LR+ of all the screening instruments (Table 3).

## 4. Discussion

The aim of the SCREEN pilot study was to determine a short-item screening tool with a good diagnostic performance to accurately identify older patients with cancer presenting signs of depression. The study compared the screening performance of the GDS-15, GDS-4, PHQ-9 and PHQ-2 to the SCID-5-S depression module A, considered as the gold standard for assessing depression in the general population. The results of the ROC analysis illustrate that the PHQ-2 has the best predictive accuracy among the screening tools explored (Figure 1).

Before the commencement of the SCREEN pilot study, the NCCN older adult oncology guidelines, recommending the use of the GDS-15 to screen for depression in older adults with cancer, were consulted. However, the clinical insight of our in-house THCWs, who routinely cover the CGA at the Kortrijk Cancer Centre, indicated that the GDS-15 may overestimate the risk of depression. Based on the need for the optimisation of the emotional status as part of the CGA, the protocol of the SCREEN pilot study was developed and approved in 2021. The NCCN only recently updated their guidelines, and the current recommendation proposes the use of the GDS-4, PHQ-9 or PHQ-2 to assess the risk of depression [23]. The outcomes of this pilot study are thus timely and in line with the most recent NCCN older adult oncology guidelines. Moreover, the results of the SCREEN pilot study show that the GDS-15 is considerably inferior to the other screening tools investigated (Figure 1).

The ELPACA cohort study recently examined the French version of the GDS-4 as a short-item screening tool for evaluating the risk of depression [24]. The authors concluded that this French GDS-4 appeared to be a clinically relevant, user-friendly tool for routine screening for depression in older patients with cancer. Despite this positive outcome, this version was not included in our analysis, as it concerns different items rather than those in the original GDS-15 questionnaire. Moreover, this version was not validated in a Belgian or Flemish patient population of older patients with cancer.

Our study population comprised older adults with cancer who were considered vulnerable based on their G8 scores. In clinical practice, it occasionally transpires that a patient may also be considered vulnerable based on the clinical insight of the THCW. The latter implies a more in-depth evaluation through a CGA despite a normal G8 score (>14/17). In the SCREEN pilot study, this applied to only one patient (Table 1). Overall, the patients were recruited at the beginning of their oncological treatment, as routine practice requires the THCW to screen the geriatric profiles at this time. The majority of the patients had been diagnosed with an advanced stage of the malignant tumour (44%) (Table 1), which could have had an impact on the study outcomes, as the patients may have appeared especially vulnerable based on the emotional assessment. While cognitive deficit was an exclusion criterion, four patients identified themselves as having cognitive decline (8%). However, this was countered by performing an additional Mini-Mental State Examination (MMSE), concluding that none of the patients included were experiencing serious cognitive deficits. Based on the patient characteristics (Table 1), we can conclude that the majority of the patients were on polypharmacy (58%), were malnourished (58%) and had a low performance status (52%). These patient characteristics should be taken into account when considering the current results in regard to the wider patient population of older adults with cancer.

A limitation of the pilot study is that the inclusion criteria were very broadly defined in the study protocol. In the implementation of the pilot, some practical considerations were necessary. For instance, unexpectedly, the treatment that the patients received after screening played a major role. As such, a large number of patients (n = 26) could not be included because it was not practically feasible. For example, those who received only a follow-up consultation after surgery and no further systemic treatment could not be approached by the research associate within the 2-week timeframe. Despite the clear workflow practiced at the oncogeriatric department of the Kortrijk Cancer Centre, it was more feasible to recruit patients who received active systemic treatment at the oncology day clinic. This is a limitation that it is important to consider in future work.

During the inclusion period of the pilot, there was a continued impact of COVID-19. This impacted upon the rate of inclusion, and there may have also been potential effects on the psychosocial well-being of the patients that should be considered [25,26]. This should be taken into account when interpreting the study results and depressive symptoms identified.

The results indicated that approximately one in five patients showed depressive symptoms based on the SCID-5-S depression module A clinical interview. This number calls for prompt intervention and treatment, ideally after the initial screening. In the SCREEN pilot study, we outlined the acceptable levels of sensitivity and specificity as 80%. The Youden index was also determined to verify an optimal cut-off score, compared with the visual inspection of ROC curves [27]. The analysis showed that a cut-off score of 2 or more achieves the best results when screening with the PHQ-2 for depression in older adults with cancer (Table 3). The suggestion that a cut-off of 2 rather than 3 may be more operational depending on the population studied has also been made in the literature [21]. Following these outcomes, we propose validating the PHQ-2 with a cut-off of 2 for use in populations of older patients with cancer. The PHQ-2 with a cut-off of 2 identified one in ten patients as not having depressive symptoms when this was not the case (false negative). The PHQ-9, on the other hand, detects that everyone may potentially be at risk of depression, as it has 100% sensitivity, including the occurrence of many false positives (n = 24). This brings us to the consideration that one must always account for regarding screening tools: a high sensitivity requires a higher clinical effort so as to further evaluate and distinguish the true positive cases who might benefit treatment. Therefore, we would opt for the PHQ-2, as it is an ultrashort screening tool with several distinct advantages promoting its use. The inclusion of a brief screening tool within CGA could support clinicians in more efficiently incorporating an assessment of the emotional status into their CGA. It could prevent possible errors involved in the use of longer, albeit high-quality, screening tools, where the administrators may be inexperienced or untrained in their appropriate use. The use of a brief screening tool limited to one psychological domain, in this case depression, is easier to implement in routine care settings. Consequently, it is likely to promote equal access to psychological services. The use of the PHQ-2 may result in a more structured method of care for emotionally distressed patients who are in need of a more in-depth emotional evaluation within CGA, thereby addressing a gap in the management of depressive symptoms. The implementation of a short screening tool necessitates the requirement of adequate follow-up in the context of treatment. If no structural, professional diagnostic option is available, the screening may be considered as purposeless. The high level of sensitivity and specificity of the PHQ-2 ensures that all patients in need of further psychological assessment and support will be identified more efficiently and potentially more rapidly. Due to its brevity, it has an increased likelihood of response and requires a smaller time investment for the patients, while placing a lesser burden on clinicians’ time, rendering it feasible for adoption in busy clinics. To conclude, this short-item version is highly correlated with the PHQ-9 and is a valid and feasible alternative that can be used to screen for depressive symptoms.

## 5. Conclusions

Although a pilot study, the SCREEN study identified the PHQ-2 as a short-item screening tool that can adequately identify older patients with cancer who are at risk for depression. It is important to highlight that the PHQ-2 is not a substitute for a diagnostic interview by a mental health professional and that tailored supportive treatment might be required. We can suggest, based on these pilot results, that the PHQ-2 is a useful screening tool in the clinical setting that can facilitate the assessment of depression in older adults with cancer, especially vulnerable patients undergoing CGA. The outcomes obtained in this pilot study will be further explored in a validation study.

## Figures and Tables

**Table 1:** Patient characteristics.

Characteristics	*n* (%)
Age, years	
≥70	50 (100)
Median (IQR)	76 (73–82)
Sex	
Male	34 (68)
Female	16 (32)
Living situation	
Living alone at home	10 (20)
Living at home with a partner/family member	36 (72)
Service flat or institution	4 (8)
Professional home care	34 (68)
Cancer site	
Upper digestive tract, stomach, pancreas	9 (18)
Genitourinary	8 (16)
Haematological malignancies	9 (18)
Lung	6 (12)
Colorectal	4 (8)
Gynaecological	4 (8)
Other (head and neck, breast, skin, CNS)	10 (20)
Stage	
I	4 (8)
II	5 (10)
III	10 (20)
IV	22 (44)
Therapy	
Chemotherapy	32 (64)
Surgery	19 (38)
Radiotherapy	13 (26)
Hormone therapy	3 (6)
Immunotherapy or targeted therapy	9 (18)
Charlson comorbidity index	
Range	0–37
Median (IQR)	1 (0–2)
Number of other daily medications	
≥5 ^1^	29 (58)
Median (IQR)	7 (4–9)
ECOG PS	
Range	0–5
Score > 1 ^2^	26 (52)
Median (IQR)	2 (1–2.75)
G8 score	
Range	0–17
Cut-off ≤ 14 ^3^	49 (98)
Median (IQR)	13 (11–14)
ADL score	
Range	6–24
Cut-off > 6 ^4^	23
Median (IQR)	6 (6–8)
iADL score	
Range 0–8	0–8
Cut-off < 8 ^5^	36
Median (IQR)	6 (4–8)
History of falls in the past year	12 (24)
CDT	
Range	0–7
Cut-off < 4 ^6^	4 (8)
Median (IQR)	7 (5–7)
GDS-15	
Range	0–15
Cut-off ≥ 5 ^7^	8
Median (IQR)	2 (1–3.375)
MNA-SF	
Range	0–14
Cut-off < 12 ^8^	29 (58)
Median (IQR)	11 (10–12.75)

Abbreviations: ADL: activities of daily living; CDT: clock drawing test; CNS: central nervous system; ECOG PS: Eastern Cooperative Oncology Group Performance Status; GDS-15: 15-item version of the Geriatric Depression Scale; iADL: instrumental activities of daily living; IQR: interquartile range; MNA-SF: Mini-Nutritional Assessment—Short Form. ^1^ A medication use score of 5 or more indicates polypharmacy. ^2^ A score of 2 or higher indicates a low performance status. ^3^ A cut-off score of 14 on the G-8 indicates vulnerability. ^4^ A cut-off score of 7 on the ADL indicates dependence. ^5^ A cut-off score of 7 on the iADL indicates dependence. ^6^ A cut-off score of 3 on the CDT indicates cognitive deficits. ^7^ A cut-off score of 5 on the GDS-15 indicates depressive symptoms. ^8^ A cut-off score of 11 on the MNA-SF indicates (risk of) malnutrition.

**Table 2:** Area under the ROC curve.

	AUROC (95% CI)
GDS-15	62.5 (42.8–82.2)
GDS-4	71.7 (55.3–88.1)
PHQ-9	87.0 (77.2–96.8)
PHQ-2	90.1 (81.4–98.8)

Abbreviations: ROC: receiver operating characteristic curve; GDS-15: 15-item version of the Geriatric Depression Scale; GDS-4: 4-item version of the Geriatric Depression Scale; PHQ-9: 9-item version of the Patient Health Questionnaire; PHQ-2: 2-item version of the Patient Health Questionnaire.

**Table 3:** Scale properties used to screen for depression.

	Sensitivity	Specificity	FP	FN	PPV	NPV	LR+	LR−	J
GDS-15	22.2%	85.4%	14.6%	77.8%	25.0%	83.3%	1.5	0.9	0.1
GDS-4									
Cut-off 1	88.9%	56.1%	43.9%	11.1%	30.8%	95.8%	2.0	0.2	0.5
Cut-off 2	11.1%	92.7%	7.3%	88.9%	25.0%	82.6%	1.5	1	0.0
PHQ-9	100.0%	63.4%	36.6%	0.0%	37.5%	100.0%	2.7	0	0.6
PHQ-2									
Cut-off 2	88.9%	87.8%	12.2%	11.1%	61.5%	97.3%	7.3	0.1	0.8
Cut-off 3	55.6%	90.2%	9.8%	44.4%	55.6%	90.2%	5.7	0.5	0.5

Abbreviations: FN: false negatives; FP: false positives; GDS-15: 15-item version of the Geriatric Depression Scale; GDS-4: 4-item version of the Geriatric Depression Scale; J: Youden index; LR−: negative likelihood ratio; LR+: positive likelihood ratio; NPV: negative predictive value; PHQ-9: 9-item version of the Patient Health Questionnaire; PHQ-2: 2-item version of the Patient Health Questionnaire; PPV: positive predictive value.

## Data Availability

The data presented in this study are available on request from the corresponding author. The data are not publicly available due to restricted access to the database.

## Appendix A

**Table A1:** Content of the comprehensive geriatric assessment (CGA).

Domain	Description	Assessment tool
Functional status	Assessment of limitations in physical function activities	Katz’s ADL * [28]
	Assessment of the ability to complete activities required to maintain independence	Lawton’s iADL ** [29]
Physical status	Number of falls in the past 12 months	Not applicable
Social status	Evaluation of living conditions, marital status, education, professional homecare	Not applicable
Cognition	Assessment of the patient’s cognitive performance	Freund CDT [30], MMSE [31]
Emotional status	Evaluation of the risk of depression	GDS-15 [32]
Nutritional status		MNA-SF [33]
Comorbidities	Assessment of the number of co-existing morbidities, rating accordingly	CCI [34]
Polypharmacy	Assessment of the number of medications	Not applicable

* The Katz ADL scale includes six items (bathing, dressing, toileting, transferring, continence and feeding). ** The Lawton IADL scale includes eight items (ability to use the telephone, shopping, cooking, housekeeping, doing laundry, responsibility for own medication, mode of transportation and ability to handle finances). Abbreviations: ADL: activities of daily living; iADL: instrumental activities of daily living; CDT: clock drawing rest; MMSE: Mini-Mental State Examination; GDS-15: 15-item version of the Geriatric Depression Scale; MNA-SF: Mini-Nutritional Assessment—Short Form; CCI: Charlson comorbidity index.

**Table A2:** The Geriatric Depression Scale—15 item version.

Choose the best answer for how you have felt over the past week.	
**1. Are you basically satisfied with your life? ***	YES/NO
2. Have you dropped many of your activities and interests?	YES/NO
**3. Do you feel that your life is empty? ***	YES/NO
4. Do you often get bored?	YES/NO
5. Are you in good spirits most of the time?	YES/NO
**6. Are you afraid that something bad is going to happen to you? ***	YES/NO
**7. Do you feel happy most of the time? ***	YES/NO
8. Do you often feel helpless?	YES/NO
9. Do you prefer to stay at home, rather than going out and doing new things?	YES/NO
10. Do you feel you have more problems with memory than most?	YES/NO
11. Do you think it is wonderful to be alive now?	YES/NO
12. Do you feel pretty worthless the way you are now?	YES/NO
13. Do you feel full of energy?	YES/NO
14. Do you feel that your situation is hopeless?	YES/NO
15. Do you think that most people are better off than you are?	YES/NO

* These questions present the four items of the GDS-4, developed by D’Ath et al. [12].

**Table A3:** The Patient Health Questionnaire.

Over the Last 2 Weeks, How Often Have You Been Bothered by Any of the Following Problems?				
	Not at All	Several Days	More than Half the Days	Nearly Every Day
**1. Little interest or pleasure in doing things ***	0	1	2	3
**2. Feeling down, depressed, or hopeless ***	0	1	2	3
3. Trouble falling or staying asleep or sleeping too much	0	1	2	3
4. Feeling tired or having little energy	0	1	2	3
5. Poor appetite or overeating	0	1	2	3
6. Feeling bad about yourself or that you are a failure or have let yourself or your family down	0	1	2	3
7. Trouble concentrating on things, such as reading the newspaper or watching television	0	1	2	3
8. Moving or speaking so slowly that other people could have noticed. Or the opposite—being so fidgety or restless that you have been moving around a lot more than usual	0	1	2	3
9. Thoughts that you would be better off dead or of hurting yourself	0	1	2	3

* These questions present the two items of the PHQ-2, developed by Kroenke et al. [14].
